# Microbiome–Genome Crosstalk in Colorectal Cancer: Colibactin Signatures and *Fusobacterium nucleatum* in Epidemiology, Driver Selection, and Translation

**DOI:** 10.3390/ijms27042068

**Published:** 2026-02-23

**Authors:** Sungwon Jung

**Affiliations:** 1 Department of Genome Medicine and Science, Gachon University College of Medicine, Incheon 21565, Republic of Korea; sjung@gachon.ac.kr; Tel.: +82-32-458-2740; 2 Gachon Institute of Genome Medicine and Science, Gachon University Gil Medical Center, Incheon 21565, Republic of Korea

**Keywords:** colibactin, mutational signatures, microbiome–genome crosstalk, colorectal cancer, *Fusobacterium nucleatum*, molecular epidemiology, precision oncology

## Abstract

Colibactin, a genotoxin produced by *pks*^+^ *E. coli*, imprints highly specific mutational signatures SBS88 and ID18 in colorectal cancer (CRC) and even in normal colonic crypts. Population-scale analyses show these signatures are enriched in early-onset CRC, vary geographically, and are imprinted early during tumor evolution, where probabilistic attribution indicates that colibactin contributes to a measurable fraction of *APC* driver mutations in colibactin-positive cancers. Beyond colibactin, *Fusobacterium nucleatum* exerts clade-specific effects on tumor ecology and therapy response, with data supporting both chemoresistance and sensitization to anti-*PD-1* in microsatellite stable (MSS) CRC. This article covers mechanistic, genomic, and molecular epidemiology evidence, outlines analytic standards for signature detection (whole-genome sequencing (WGS)/whole-exome sequencing (WES), single-sample fitting, and limits at low mutation counts), and charts translational paths spanning noninvasive screening (stool metagenomics + mutational signatures in tissue/circulating tumor DNA (ctDNA)), risk stratification, and microbial-targeted interventions (antibiotics, phages, *ClbP* inhibitors). Framing microbiome–genome crosstalk as a tractable axis enables testable clinical hypotheses for precision oncology.

## 1. Introduction

CRC remains a major global health burden as the third-most diagnosed malignancy and the second leading cause of cancer-related mortality worldwide [1]. In 2020 alone, over 1.9 million new CRC cases were diagnosed and about 935,000 deaths occurred. While incidence and mortality have stabilized or declined in some high-resource regions due to screening and risk factor modifications, and an alarming trend has emerged: CRC rates are rising among adults under 50 years of age across diverse countries [2]. Recent analyses of cancer registry data from 50 countries found that early-onset CRC incidence (ages 25–49) is increasing in 27 of them, often outpacing or occurring in contrast to stable rates in older adults. This global phenomenon of “young-onset” CRC—now accounting for nearly one in ten new cases—has prompted intense investigation into its causes, with attention turning to contemporary lifestyle and environmental exposures that might accelerate colorectal tumorigenesis in younger cohorts. Accumulating evidence implicates perturbations of the gut microbiome as a key contributor to these epidemiologic shifts [3]. The human intestinal microbiome, containing trillions of microbes and their collective genomes, sits at the interface between environment and host and is increasingly recognized as an active modulator of CRC risk and behavior [3].

Microbiome–genome crosstalk in CRC has become a focal point of research, referring to the dynamic interactions by which gut microorganisms can influence host genetic pathways, induce mutations, and shape tumor biology [3]. Bacteria in the colon can produce metabolites and toxins that damage DNA or alter cellular signaling, thereby directly affecting colorectal carcinogenesis. A conceptual framework known as the “driver-passenger” model encapsulates this idea: certain microbial species act as tumor-initiating *drivers* by creating a pro-carcinogenic microenvironment (through genotoxin production, inflammation, or DNA damage), after which other opportunistic bacteria (*passengers*) colonize established tumors and may further influence cancer progression [3]. Consistent with this model, multiple bacterial taxa have been implicated in CRC development, each via distinct mechanisms. Notably, *Escherichia coli* strains harboring the polyketide synthase (pks) island and the oral anaerobe *Fusobacterium nucleatum* have emerged as two key players in CRC pathogenesis. These microbes exemplify how the gut flora can affect tumor initiation and behavior. *Pks*^+^ *E. coli* produces a genotoxin metabolite that directly mutates the colonic epithelium, whereas *F. nucleatum* remodels the tumor immune microenvironment and modulates therapy responses.

Certain *E. coli* within the gut carries a symbiotic plasmid-borne gene cluster (the *pks* island) that encodes colibactin, a small molecule genotoxin. Colibactin can cause DNA double-strand breaks and interstrand crosslinks in host cells [4,5]. Recent studies have shown that this bacterial toxin leaves a characteristic imprint on the cancer genome in the form of two highly specific mutational signatures. A single-base substitution pattern (SBS88) and an insertion/deletion pattern (ID18) [4]. These signatures were first identified in human intestinal organoid experiments with *pks*^+^ *E. coli* and then recognized in subsets of human CRC genomes [5]. Population-scale WGS analyses further indicate that SBS88/ID18 are enriched in early-onset CRC and are often truncal, consistent with early imprinting and potential effects on driver selection (notably *APC*). Colibactin’s mutational footprint has also even been detected in normal colonic epithelium adjacent to tumors [5], suggesting that exposure in normal tissue can precede malignant transformation. Together, these findings position the *pks*^+^ *E. coli*–colibactin axis as a paradigm for microbiome-induced metagenesis in CRC—a literal microbial signature written into the tumor genome [4,5].

In addition to direct mutagenic effects, gut bacteria can profoundly influence tumor behavior through immune modulation and microenvironmental changes. *Fusobacterium nucleatum* (an oral commensal often found enriched within colorectal tumors) has emerged as a central example of how bacteria shape the colorectal tumor immune landscape. *F. nucleatum* is frequently detected in colorectal adenomas and cancers, with studies showing significantly higher *Fusobacterium* abundance in adenomatous tissue and CRC stool samples compared to normal controls [6]. In *Apc*^Min/+^ mouse models, introducing *F. nucleatum* accelerates intestinal tumor formation and selectively recruits tumor-infiltrating myeloid cells (such as tumor-associated neutrophils and macrophages) into the tumor stroma. This bacterium effectively creates a proinflammatory microenvironment that can foster immune evasion and tumor growth. Unlike colibactin-producing *E. coli*, *F. nucleatum* is not thought to induce mutations directly. Instead, it promotes cancer by altering cell signaling and immunity. *F. nucleatum* can adhere to and invade colonic epithelial cells (for example, via its *FadA* adhesin binding E-cadherin), activating β-catenin and inflammatory pathways. It also expresses virulence factors (like *Fap2*) that interact with immune cell receptors to dampen anti-tumor immunity. Clinically, the presence of *F. nucleatum* in CRC tissue has been correlated with aggressive tumor features and poorer patient prognosis in several studies [6]. Notably, *F. nucleatum* has been implicated in resistance to chemotherapy. It was observed that CRC patients who developed recurrence after 5-fluorouracil-based chemotherapy had higher intratumoral *Fusobacterium*, and mechanistic work revealed that *F. nucleatum* infection can induce chemoresistance by activating Toll-like receptor-4 (*TLR4*) and *MYD88* signaling, which in turn triggers pro-survival autophagy in cancer cells [7]. By switching stressed tumor cells from apoptotic death to an autophagic state, *F. nucleatum* helps tumors withstand chemotherapeutic stress. Paradoxically, recent research also suggests that *F. nucleatum* may in some contexts sensitize tumors to immunotherapy. In a 2021 study, high levels of *Fusobacterium* in MSS CRC were associated with improved responses to anti-*PD-1* checkpoint inhibitors [8]. *F. nucleatum* was shown to activate the stimulator of interferon genes (STING) pathway in tumor cells and increase infiltration of *IFN*-γ^+^ *CD8*^+^ T cells under *PD-1* blockade, thereby augmenting the anti-tumor immune response. These findings highlight that the impact of *F. nucleatum* on tumor immunity and therapy can be context-dependent or even strain-specific (with different *Fusobacterium* clades potentially exerting different effects). Nonetheless, they emphasize a broader principle: The microbiome can condition the tumor microenvironment in ways that significantly alter disease course and treatment outcomes [6,7,8].

**Figure 1:**
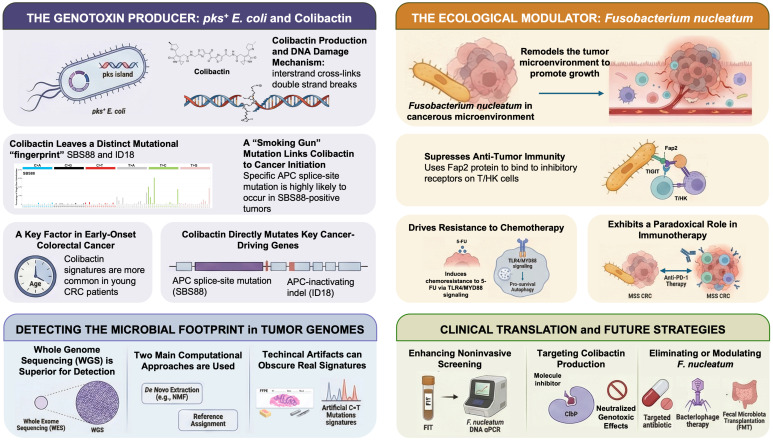
Conceptual overview of microbiome–genome crosstalk in CRC that is covered in this review. Colibactin from *pks^+^ E. coli* induces DNA lesions that are fixed as the mutational signatures SBS88 and ID18, which are enriched in specific epidemiological contexts and can contribute to early driver selection (e.g., *APC*). In parallel, Fn shapes the tumor microenvironment and modulates therapy response. It also highlights translational opportunities for combining microbial assays and host genomic signatures for screening, stratification, and microbiome-targeted interventions.

In the following sections, we comprehensively examine the multifaceted crosstalk between the gut microbiome and the colorectal cancer genome, with an emphasis on the two prototypical microbes introduced above—colibactin-producing *E. coli* and *F. nucleatum*. We assess mechanistic evidence of how these bacteria (and others) contribute to colorectal carcinogenesis at the molecular level, from DNA damage and mutational driver selection to immune evasion and metastatic signaling. We also review the emerging genomic and molecular epidemiologic data linking microbiome factors to distinct mutational signatures and tumor subtypes (for example, differences in microbial exposure that may explain early-onset CRC trends or geographic incidence patterns). In doing so, we discuss current analytical approaches and standards for detecting microbiome-induced genomic changes, including the bioinformatic identification of mutational signatures (SBS/ID) from tumor sequencing data and the challenges of attributing causation in single samples. Finally, we highlight promising translational applications that arise from this microbiome–genome interplay. These include strategies for noninvasive CRC screening by combining stool microbiome profiles with mutation signature analysis, risk stratification tools that incorporate microbial exposure history, and novel therapeutic avenues such as targeting microbiome genotoxins (e.g., *ClbP* inhibitors to block colibactin) or modulating tumor-associated microbes to improve immunotherapy responses. By integrating insights across basic, population, and clinical research, this review aims to clarify how microbial organisms can shape the genomic landscape of CRC and to illustrate how this knowledge can be harnessed for improved cancer prevention and precision oncology. A schematic overview of the microbiome–genome crosstalk framework and the translational roadmap discussed in this review is provided in Figure 1.

## 2. Colibactin-Induced Mutational Signatures in CRC

### 2.1. Colibactin Production by pks^+^ E. coli—Mechanism of Genotoxicity

Certain *Escherichia coli* strains harbor a 54 kb genomic island, known as *pks* (genes *clbA*-*clbS*), that encodes a multi-enzyme assembly line for colibactin biosynthesis [9,10]. Colibactin is a diffusible small molecule genotoxin implicated in CRC development [11,12]. The *pks* island encodes a hybrid polyketide synthase/non-ribosomal peptide synthetase (*PKS*/*NRPS*) pathway and accessory enzymes that together produce colibactin [9]. A key enzyme is *ClbA*, a 4′-phosphopantetheinyl transferase (PPTase) that post-translationally activates the *PKS*/*NRPS* proteins by adding phosphopantetheine arms to their carrier domains. The *PKS* and *NRPS* modules (*ClbC*, *ClbO*, *ClbH*, *ClbJ*, *ClbN*, etc.) sequentially assemble an inert precursor, precolibactin, which carries a unique N-myristoyl-D-asparagine prodrug motif at one end [9,13]. A thioesterase enzyme, *ClbQ*, then releases intermediate polyketide-peptide chains from the assembly line, after which the completed precolibactin is exported to the periplasm by the *ClbM* transporter [9]. In the periplasm, a peptidase called *ClbP* cleaves off the N-myristoyl-D-Asn “blocking group”, thereby activating the molecule as colibactin [9,13]. The activated colibactin is subsequently released from the bacterium (the exact export mechanism beyond the periplasm is still unclear) [9]. Notably, colibactin’s final structure is highly unstable and was only deduced in 2019 via biosynthetic trapping and total synthesis [13]. It is a complex molecule featuring two fused macrocyclic dihydropyridone rings and two electrophilic cyclopropane rings—these cyclopropane moieties serve as the DNA-reactive “warheads” that alkylate host DNA [13,14].

To avoid self-inflicted genotoxicity, colibactin-producing bacteria employ protective mechanisms. First, the above-mentioned prodrug motif (cleaved by *ClbP*) likely keeps colibactin inactive until it reaches the periplasm [9]. Second, the bacteria produce *ClbS*, a dedicated resistance enzyme that inactivates colibactin [15]. *ClbS* is a cyclopropane hydrolase that intercepts any active colibactin that diffuses back into the bacterial cytoplasm and enzymatically opens the cyclopropane rings, rendering the toxin non-genotoxic. In this way, *ClbS* detoxifies colibactin and protects the bacterial DNA from damage. Consistent with these roles, mutation of any single *clb* gene (except the expendable *clbS*) abolishes colibactin’s genotoxic effects [9,10,15]. In other words, all components of the *PKS/NRPS* pathway are essential for producing the active toxin [9].

Once secreted into host tissues, colibactin alkylates host DNA, causing covalent modifications on nucleotide bases that ultimately link the two strands of DNA [13]. The two cyclopropane rings of colibactin react with nucleophilic sites on DNA (preferentially the N3 positions of adenines on opposite strands), forming a bilateral interstrand cross-link (ICL) in the DNA duplex [4]. This cross-linking is the primary mechanism of colibactin’s genotoxicity. The ICLs prevent the DNA double helix from unzipping during replication and transcription, leading to stalled replication forks and activation of DNA damage responses. Exposed mammalian cells exhibit hallmarks of ICL-induced stress, such as *AKT* kinase activation and recruitment of the Fanconi anemia repair pathway. If the cross-links are not repaired, attempted DNA replication can convert these lesions into DNA double-strand breaks (DSBs). Accordingly, cultured cells briefly infected with *pks*^+^ *E. coli* develop DSB markers (phosphorylated γ*H2AX* foci) within hours of exposure [10]. The cellular outcome is G2/M cell-cycle arrest with *ATM* checkpoint activation, followed by either DNA repair and survival (often with mutations) or elimination of heavily damaged cells via apoptosis or senescence. Notably, adding an excess of exogenous DNA to cell cultures can “rescue” cells from colibactin toxicity by sequestering the toxin, demonstrating that colibactin’s DNA-targeting activity underlies the observed damage.

Substantial evidence links colibactin’s genotoxic mechanism to colorectal carcinogenesis. Transient infection of human cell lines with colibactin-producing *E. coli* causes DNA DSBs and chromosomal abnormalities. Chronic exposure leads to genomic instability (aneuploidy) and even cellular transformation in vitro [10]. In animal models, colonization of the colon with *pks^+^ E. coli* induces DNA damage in gut epithelial cells and significantly promotes colorectal tumor formation, especially in the context of pre-existing inflammation [11]. For example, in *Il10*^−/−^ mice treated with a carcinogen, only *E. coli* with an intact *pks* island can drive invasive CRC, whereas isogenic Δ*pks* mutants show markedly reduced tumorigenicity. Similarly, introducing *pks^+^ E. coli* into conventional or germ-free mouse models enhances intestinal tumor burden compared to non-genotoxic (Δ*pks*) strains. Consistent with these experimental findings, *pks^+^ E. coli* are over-represented in the gut microbiota of CRC patients [12]. While only ~20% of healthy individuals carry *pks*-harboring *E. coli*, about 60–70% of CRC patients have detectable *pks^+^ E. coli* in their colon tissue or stool, indicating a strong association between colibactin-producing bacteria and human colorectal tumors. Most strikingly, recent whole-genome sequencing studies have identified a distinctive mutational “fingerprint” in human colorectal tumors that is attributable to past colibactin exposure [4,16]. This signature—characterized by specific base-substitution and indel mutations—was first reproduced by exposing human intestinal organoids to *pks^+^ E. coli* and then observing the resulting mutations upon subsequent organoid expansion [16]. Approximately 5–10% of sporadic CRCs harbor this colibactin-associated mutational signature [4,16]. Together, these insights establish colibactin as a key mediator of microbiome–genome crosstalk in CRC—a microbial metabolite that hijacks host DNA, inflicts cross-links and strand breaks, and can thereby initiate tumorigenic mutations and genomic instability in colonic cells [13,16].

### 2.2. Colibactin-Associated Mutational Signatures—Identification and Features

Colibactin leaves a characteristic imprint on the tumor genome. Mutational signature SBS88 (a single-base substitution pattern) and ID18 (a small insertion/deletion pattern) have been identified as the distinctive “footprints” of colibactin exposure in CRC [16]. The first evidence came from in vitro experiments. Human colonic organoids repeatedly co-cultured with colibactin-producing *E. coli* accumulated a unique constellation of mutations that was absent in control organoids infected with an isogenic *E. coli* lacking the *pks* island [17]. Whole-genome sequencing of these organoids revealed a novel base substitution signature (dubbed “SBS-*pks*”) as well as a matching indel signature (“ID-*pks*”). Notably, this pattern closely matched an unusual mutational signature that had been observed in a small subset of human CRCs, suggesting a direct link between the bacterial exposure and tumor mutations. Subsequent analyses of large CRC cohorts have confirmed that ~5–15% of MSS colorectal cancers carry these colibactin-associated signatures [4,16]. The COSMIC database (v3) formally catalogued the signatures as SBS88 and ID18 and recognized them as markers of genotoxic *pks^+^ E. coli* activity in tumorigenesis. Key studies establishing the link between colibactin-producing *E. coli*, mutational signatures, and CRC are summarized in Table 1.

The base substitution signature SBS88 is characterized by an excess of thymine mutations in a specific sequence context [4,16]. Most SBS88 mutations are T → N substitutions (T to any other base) and occur at thymine bases that have adenines immediately upstream on the 5′ side. The flanking sequence motif is very distinctive, as analyses show a strong enrichment for adenine at the −3 and −4 positions (i.e., two and three bases 5′ of the mutated T) as well. This bias aligns with colibactin’s known reactivity—the toxin preferentially alkylates adenine residues in DNA [13], which can lead to error-prone repair or mispairing at adjacent thymine bases. As a result, SBS88 displays a unique A/T-rich sequence context not seen in common endogenous mutational processes [4,16]. The SBS88 mutational profile is dominated by T → C, T → A, and T → G changes at these A-flanked T sites [17]. When present in a tumor, SBS88 can contribute substantially to the mutation burden—often accounting for 10–30% of all single-base mutations in an SBS88-positive tumor—yet it is virtually absent in colorectal cancers lacking colibactin exposure.

Colibactin’s genomic footprint also includes a characteristic small insertion/deletion pattern, designated ID18. ID18 is defined by a predominance of single-base deletions of thymine in homopolymeric runs of T’s [4,16]. In practical terms, ID18 mostly consists of one- or two-base deletions occurring at sequences like TTT… (≥3 Ts in a row), especially when that T-run is immediately preceded by 2–4 adenines in the sequence. In other words, ID18 manifests as the loss of one or a few T’s from an A-rich poly-T tract. This mirrors the SBS88 context bias and likely results from colibactin-induced DNA cross-links or breaks at fragile T-rich sites, followed by imperfect repair that yields a contraction of the T repeat [4,13,16]. The specificity of ID18 (short T-deletions in an A/T-rich context) makes it a distinctive marker of colibactin damage—it is rarely generated by normal aging or other mutagenic processes in CRC [4,16]. Indeed, in the organoid experiments, both the SBS-*pks* substitution pattern and the ID-*pks* indel pattern emerged in colibactin-exposed organoids (and in neither the untreated nor Δ*pks* controls), confirming that colibactin induces coupled point mutations and indels in its target cells.

The discovery of SBS88 and ID18 was bolstered by direct experimental validation. In the landmark study, human colonic organoids were co-cultured with a colibactin-producing *E. coli* over five months, simulating chronic exposure [16]. Whole-genome sequencing of these organoids revealed a distinctive mutational profile—termed “SBS-*pks*” and “ID-*pks*” in that study—which matched SBS88 and ID18, respectively, in trinucleotide and indel spectra. Control organoids exposed to a non-genotoxic isogenic strain (lacking a critical colibactin gene) showed none of these mutations, proving that colibactin was the causative agent of the observed signature [16]. Additional lines of evidence have since reinforced this causation. Normal human colon epithelial cells exposed to colibactin (for instance, via controlled infections or by studying colonic crypts that naturally harbored *pks*^+^ bacteria) exhibit mutational profiles congruent with SBS88/ID18 [19]. Likewise, mouse models and cell lines have been used to verify that colibactin exposure can imprint these mutations, though much of our knowledge comes from human data. Mechanistically, the colibactin signatures seem to arise immediately during exposure. Colibactin inflicts DNA adducts that, upon DNA replication or error-prone repair, are converted into permanent mutations in the next cell generation [4]. These mutations can then be “frozen” into the genome of a clonal cell population that eventually expands into a tumor—explaining how a cancer can carry the colibactin signature long after the causal bacteria may have disappeared.

SBS88 and ID18 have distinctive features that set them apart from other CRC mutational signatures, highlighting their exogenous (microbial) origin. First, their nucleotide context biases are highly specific (favoring A/T-rich sequences), whereas ubiquitous endogenous signatures like SBS1 and SBS5 are relatively unbiased and occur broadly across the genome. SBS1, for example, reflects spontaneous deamination of 5-methylcytosine and produces C → T transitions at CpG sites in an age-dependent manner [19]. SBS1 mutations accumulate steadily with cell divisions and age and are found at comparable levels in essentially all CRCs (usually contributing 10–20% of mutations in a tumor as a “clock” signature). However, SBS1 does not preferentially target homopolymer tracts or specific flanking bases—in stark contrast to the focused A/T-targeting of SBS88. SBS5 is another ubiquitous signature of unknown etiology that manifests as a flat, randomly distributed mutation spectrum increasing with age. Like SBS1, SBS5 has no strong sequence-context preference and appears in all individuals, and it simply adds a background of mutations over time. Neither SBS1 nor SBS5 can explain the highly localized T-base changes and T-deletions that characterize colibactin’s effect [21]. On the other hand, consider SBS18, an oxidative stress signature linked to *ROS*-induced 8-oxoguanine formation. SBS18 is dominated by C → A transversions at G bases (especially at GpTpX contexts)—a pattern entirely different from the thymine-centered changes in SBS88 [21]. This emphasizes that SBS88 and ID18 arise from a distinct exogenous cause (microbial toxin) rather than from general endogenous processes like aging, chronic inflammation, or reactive oxygen species [4,16]. Importantly, when SBS88/ID18 are present in a tumor, they often co-occur alongside the common “housekeeping” signatures (like SBS1 and SBS5) that accumulate with age, but their distinct motif bias can help define and etiologically distinct subset of CRC. The implications of colibactin-linked signatures for early driver selection are discussed below (Section 2.4) [4,17].

Another notable feature of SBS88 is its timing and distribution in colorectal carcinogenesis. Studies indicate that colibactin-induced mutational damage tends to occur early in life, long before cancer diagnosis. This signature is often detectable in normal colorectal crypt cells from individuals’ years (even decades) before a tumor develops [4]. In fact, mutational analyses of normal colon tissue suggest colibactin exposure may imprint many of its mutations in the first decade of life, and that the mutagenic exposure then ceases thereafter. This early life “mutational burst” could give an exposed colon a head start of oncogenic mutations equivalent to decades of spontaneous accumulation. Consistent with this idea, SBS88 and ID18 have been found enriched in early-onset CRC cases. It has been reported that colibactin-associated signatures were 3.3 times more common in tumors from patients under 40 years old than in those over 70. Using the conventional early-onset cutoff of 50 years, a similar trend was observed—colibactin signatures were about 3–4× more frequent in CRCs diagnosed before age 50 than in later-onset cases. Moreover, colibactin-positive tumors tend to arise in slightly younger patients on average (median age ~5–10 years younger) and more often in the distal colon and rectum, compared to colibactin-negative tumors. These epidemiological patterns point to a strong link between early-life harboring of *pks*^+^ bacteria and early-onset colorectal carcinogenesis. They also align with the observation that the colibactin mutational imprint is often “truncal” in tumors—i.e., present in mutations that occur at the very beginning of tumor development. In many SBS88-positive cancers, colibactin-type mutations (e.g., certain *APC* mutations) are found in all cells of the tumor (including in metastases), indicating they arose in the initial transformed cell and were propagated through all clonal expansions [17]. Indeed, one recurrent colibactin-linked mutation (*APC*:c.835-8A>G, discussed below) has been detected even in premalignant colorectal adenoma, implying that colibactin exposure can initiate the adenoma-carcinoma sequence at the very outset [4].

### 2.3. Epidemiological Patterns of Colibactin Signatures: Geographic Variation

Geographically, the prevalence of colibactin signatures in CRC correlates with regional differences in cancer incidence and lifestyles. Populations with the highest CRC incidence rates also show the highest burdens of SBS88/ID18 in tumors [4]. In the 11-country study, mutation loads of SBS88/ID18 were significantly elevated in high-incidence regions (such as parts of Eastern Europe, Japan, and Argentina) compared to intermediate-incidence regions (like Thailand, Iran, and Brazil). Across all MSS cases in that cohort, ~21% had detectable colibactin signatures, but this proportion ranged from lows of ~5–10% in certain low-risk countries up to highs of ~30% in some high-risk countries (mirroring their CRC rates) [4]. Such variation likely reflects underlying differences in exposure to colibactin-producing bacteria, driven by factors like diet, hygiene, and early-life microbiome composition. Diet is thought to play a role because *pks^+^ E. coli* appear to thrive in westernized high-fat, low-fiber diets, and it has been found that adherence to a Western diet was associated with a higher incidence of CRCs containing *pks^+^ E. coli* [17]. Additionally, antibiotic use patterns might influence *pks E. coli* colonization—for instance, broad antibiotic exposure in childhood could either promote or inhibit the establishment of *pks* bacteria. Epidemiologically, the past few decades have seen rising early-onset CRC rates in many countries alongside changes in diet, obesity, and possibly childhood antibiotic use [4]. The colibactin mutational signature provides molecular evidence tying these trends to a microbiome-derived mutagenic exposure in early life. It is a tangible link between environment (gut microbiota) and somatic mutation in CRC, and it helps explain why certain regions and birth cohorts have experienced unusual upticks in colorectal cancer incidence.

### 2.4. Influence on CRC Driver Mutations

One of the most significant implications of colibactin exposure is its impact on driver mutations in colorectal cancer—particularly mutations in the *APC* tumor suppressor gene, which is the gatekeeper of colorectal tumor initiation. Consistent with the SBS88/ID18 motif preference described above (Section 2.2), *APC* driver mutations are enriched among colibactin-attributable events in signature-positive tumors [4,16]. Notably, roughly 5% of all *APC* mutations in CRC appear to carry the colibactin-associated sequence context [17]. In tumors that are positive for the colibactin signature, the proportion is even greater. Probabilistic assignment models indicate that colibactin exposure accounts for a substantial share of *APC* hits in those cases [4,17]. For example, colibactin-signature motifs were identified in 15.5% of all *APC* driver mutations in colibactin-positive cancers (versus only ~1% of *APC* mutations in signature-negative cancers) [17]. Similarly, it has been found that over 20% of tumors with high SBS88/ID18 activity harbored an *APC* mutation that was plausibly induced by colibactin (based on sequence context) [17]. These numbers emphasize that colibactin exposure can be a direct contributor to *APC* inactivation during colorectal tumorigenesis.

A striking example is the recurrent *APC* splice-site mutation c.835-8A>G. This mutation (an A → G substitution at an intronic position 8 based upstream of exon 9) disrupts normal splicing of *APC* and produces a truncated protein [20]. Intriguingly, the mutated adenine lies in an “ATT” sequence context—exactly the 5′-T motif favored by SBS88 [17]. It has been shown that *APC*:c.835-8A>G is hugely enriched in colibactin-positive tumors. 83.5% of CRCs harboring *APC*:c.835-8A>G showed the colibactin mutational signature, compared to <17% of cancers lacking the signature. Statistically, this *APC* splice mutation was 65 times more likely to occur in SBS88-positive tumors than in SBS88-negative tumors (odds ratio ~65, *p*~3 × 10^−80^). Such an extreme association strongly suggests a causal link that *APC*:c.835-8A>G is essentially a colibactin imprint in the tumor genome [17,20]. Indeed, this mutation was first noted in patients with unexplained early-onset polyposis (numerous colonic adenomas without a germline *APC* mutation). Genomic studies of those patients’ tumors revealed the colibactin mutational signature and the *APC*:c.835-8A>G change together [4], implying chronic colonization by *pks^+^ E. coli* as an etiological factor in their disease [20]. And *APC*:c.835-8A>G emerged as the single most characteristic mutation of colibactin exposure, to the point that its presence in a tumor can be used as a proxy indicator of past colibactin activity [17].

Beyond *APC*:c.835-8A>G, several other driver mutations have been statistically linked to colibactin [17]. Another *APC* splice-site variant, c.1549-8A>G, occurs predominantly in SBS88-positive tumors (90% of cases with *APC*:c.1549-8A>G were colibactin-signature positive) [17]. Likewise, the recurrent nonsense mutation *APC*:p.Lys534* (c.1600A > T) is significantly enriched in colibactin-exposed cancers (70% of cases with this mutation had SBS88) [17]. In addition to SBS88-type substitutions, ID18-associated frameshift deletions contribute substantially to *APC* truncation events in signature-positive tumors. One analysis estimated that ~25% of all *APC* truncating indels in colibactin-positive cancers are attributable to ID18 [4]. Together, these data support a model in which colibactin can accelerate the earliest step of colorectal mutagenesis by promoting *APC* inactivation.

Timing analyses indicate that colibactin-induced driver mutations often occur very early in tumor evolution. The *APC*:c.835-8A>G mutation, for instance, is usually found at a high variant allele fraction (VAF) in tumors, meaning it is present in essentially all cancer cells of that tumor—consistent with it being a founding clonal mutation. SBS88-positive cancers showed that the VAF of *APC*:c.835-8A>G is on average higher than that of other mutations in the same tumors, suggesting it likely occurred in the tumor-initiating cell and was present in all subsequent clones [17]. More broadly, SBS88-type mutations in CRC tend to be truncal (shared by all regions of a tumor) rather than subclonal, implying they were acquired before the malignant clone began diversifying [4,17]. This is exemplified in cases where primary tumors and matched metastases both carry the colibactin signature—indicating the mutational damage that happened prior to metastasis and likely at the adenoma stage. As mentioned, colibactin-like mutational patterns have even been detected in normal colon crypts of cancer patients, especially in crypts adjacent to tumors [19]. Lee-Six et al. (2019) [18] had earlier reported two unknown signatures in normal colon cells that appeared only in early childhood, and these were later recognized to match SBS88 and ID18, pointing to childhood exposure to *pks* bacteria [4]. Furthermore, the *APC*:c.835-8A>G mutation has been observed in benign adenomatous polyps that carry the colibactin signature, reinforcing that colibactin exposure can initiate the first hit in *APC* even at the pre-cancerous stage [18,20].

While *APC* is the most prominent target of colibactin’s mutagenesis, other CRC driver genes show similar vulnerability. A handful of recurrent mutations have been identified in non-*APC* drivers that are highly enriched in colibactin-signature positive tumors [17]. For example, a parallel splice-site mutation in *SMAD4* (c.788-8A>G) is almost exclusively found in SBS88-positive cancers (86% of cases with *SMAD4*:c.788-8A>G had the colibactin signature) [17]. This mutation, like *APC*:c.835-8A>G, occurs at an adenine in an “ATT” context, fitting the SBS88 motif. Another is a hotspot missense mutation in *TP53*—p.Tyr220Cys (c.659A>G)—which is significantly overrepresented in colibactin-positive CRCs (odds ratio (OR) ~5.9, *p*~3 × 10^−5^). *TP53*^Y220C^ is a known driver change, and the fact that it appears frequently in SBS88 tumors suggests colibactin can occasionally induce classic oncogenic mutations (not just truncations) if the sequence context is favorable. Similarly, an oncogenic mutation in *PIK3CA*—p.Met1043Val (c.3127A>G)—has been noted almost exclusively in colibactin-signature cancers. All these mutations share the colibactin motif (adenines flanked by thymidines). More broadly, an excess of colibactin-signature mutations has been observed in genes like *ARID1A*, *TCF7L2*, *TGFBR2* and other drivers in SBS88-positive tumors, especially those genes that are commonly mutated early in CRC development [19]. This pattern indicates that colibactin exposure can shape the driver landscape of a tumor by “favoring” certain mutations that its mechanism produces readily. It effectively means the microbiome (via colibactin) is contributing to which driver mutations occur, and when they occur, during colorectal carcinogenesis.

In summary, colibactin-induced mutational signatures not only serve as a forensic marker of past exposure but also have direct consequences on tumor biology through their influence on driver genes. These signatures SBS88 and ID18 highlight a subset of CRCs in which a microbial toxin accelerated the accumulation of tumor-initiating mutations (notably in *APC*). The recurrent, signature-linked mutation (*APC* splice-site lesions, frameshifts, etc.) emphasize a model where a genotoxic bacterium in the gut microbiome leaves a lasting imprint on the host genome, “guiding” the tumor’s evolutionary path. This has implications for prevention and therapy. For instance, individuals with colibactin-signature mutations might benefit from early screening or from interventions targeting *pks E. coli* in their microbiome. More immediately, recognizing the colibactin signature in a tumor can inform researchers about its etiology and might prompt therapeutic exploration of synthetic lethal vulnerabilities (e.g., exploiting the tumor’s DNA repair deficiencies). Colibactin’s impact on *APC* and other drivers is a vivid example of how bacteria can directly contribute to cancer-causing mutations—cementing the concept of a microbially driven pathway to colorectal cancer alongside the traditional environmental and hereditary factors [4,16,17].

## 3. *Fusobacterium nucleatum* in Tumor Ecology and Therapy Response

### 3.1. Fusobacterium nucleatum and the Tumor Microenvironment

*Fusobacterium nucleatum* (Fn) is frequently enriched within colorectal tumors and functions as an active ecological component of the tumor microenvironment rather than a passive bystander. Experimental work in the *Apc*^Min/+^ model established a causal link between Fn colonization and tumor promotion. Fn administration accelerated intestinal tumorigenesis and was associated with increased infiltration of myeloid immune cells and induction of inflammatory gene programs within tumors [6]. These findings align with the concept that Fn can support a “smoldering” inflammatory niche within tumors without necessarily producing overt colitis, implying that localized immune remodeling—rather than generalized intestinal inflammation—can be sufficient to facilitate tumor progression [6].

In human CRC, intratumoral Fn abundance has been repeatedly associated with altered immune contexture and clinical behavior. Tissue-based studies have reported that Fn-high tumors can show reduced tumor-infiltrating lymphocytes, particularly *CD3*^+^ and cytotoxic *CD8*^+^ T cells, and these immune features correlate with poorer outcomes in some cohorts [22]. More recent analyses further suggest that Fn-positive tumors can exhibit a shift toward immunosuppressive or dysfunctional T-cell states—such as enrichment of exhausted *PD-1*^high^ *CD8*^+^ T cells and *FoxP3*^+^ regulatory T cells—consistent with impared anti-tumor immune surveillance [23].

Mechanistically, Fn engages innate immune pathways that promote tumor-supportive inflammation. Fn ligands can activate pattern-recognition receptors on epithelial cells and immune cells, including *TLR4*, leading to downstream *NF-kB* signaling and induction of pro-inflammatory mediators [24]. In CRC models, Fn-driven *TLR4—MyD88—NF-kB* activation has been linked to tumor-promoting transcriptional programs, including upregulation of oncogenic *microRNA-21* and cytokine networks that support proliferation, survival, invasion, and angiogenesis [24]. In parallel, Fn can directly activate epithelial oncogenic signaling. The Fn adhesin *FadA* binds E-cadherin and activates β-catenin signaling, promoting proliferative and inflammatory responses that are consistent with enhanced carcinogenesis [25]. These epithelial programs likely feedback on the immune ecosystem by altering chemokine production and antigen presentation, thereby shaping immune cell recruitment and function.

Fn also exerts immune-evasive effects through direct interference with cytotoxic lymphocytes. A well-established mechanism involves the outer membrane protein *Fap2*, which binds the inhibitory receptor *TIGIT* on NK cells and T cells, suppressing cytotoxic activity and enabling tumor cells to evade immune killing [26]. In addition, Fn expresses other immunomodulatory factors; for example, the adhesin *RadD* has been reported to engage inhibitory receptors on NK cells (e.g., *Siglec-7*), further dampening NK cell anti-tumor activity [27]. Beyond receptor–ligand inhibition, Fn can leverage tumor-associated glycans to colonize tumor tissue. *Fap2*-mediated binding to tumor-expressed *Gal-GalNAc* promotes Fn enrichment within CRC, thereby sustaining continuous microbial–immune interactions in situ [28].

Collectively, these findings support a model in which Fn contributes to CRC progression by (i) amplifying localized innate inflammatory signaling (e.g., *TLR4/NF-kB*), (ii) activating epithelial oncogenic pathways (e.g., E-cadherin/β-catenin), and (iii) suppressing effective adaptive immunity via direct inhibitory receptor engagement and broader immune dysfunction [6,24,25,26,27]. This Fn-shaped tumor ecology provides a mechanistic foundation for understanding why Fn is frequently associated with aggressive tumor phenotypes and motivates strategies at disrupting Fn-host interactions to restore anti-tumor immunity.

### 3.2. Fusobacterium nucleatum and Therapy Response

Fn has emerged as an important modifier of therapy response in CRC, with evidence spanning clinical correlations, mechanistic studies, and preclinical intervention models. The most consistent association has been with chemoresistance, particularly to 5-fluorouracil (5-FU)-based regimens. In patients, higher Fn levels in tumor tissue have been linked to recurrence after chemotherapy and poorer therapeutic outcomes [7]. Mechanistically, Fn can protect CRC cells from chemotherapy-induced death by engaging pro-survival programs. A study demonstrated that Fn activates *TLR4—MyD88* signaling in tumor cells, downregulating microRNAs (including miR-18a* and *miR-4802*) that normally restrain autophagy, and this shift promotes autophagy-dependent survival under 5-FU and oxaliplatin exposure [7]. In parallel, Fn can increase expression of anti-apoptotic factors—most notably *BIRC3* (*clAP2*)—via *TLR4/NF-kB* signaling, thereby reducing caspase-mediated apoptosis and decreasing chemotherapy efficacy in vitro and in vivo [29].

Recent work suggests that Fn-associated resistance extends beyond apoptosis to other forms of regulated cell death. Fn has been reported to suppress chemotherapy-induced pyroptosis through a *Hippo—YAP—BCL-2* axis that limits *GSDME*-mediated membrane pore formation and inflammatory cell death [30]. Fn has also been implicated in dampening ferroptosis through signaling that increases expression of lipid peroxide repair machinery (e.g., *GPX4*) downstream of epithelial adhesion/β-catenin-associated pathways, thereby promoting tumor cell survival under oxaliplatin stress [31]. Together, these studies support a multi-layered model of Fn-mediated chemoresistance in which autophagy activation and suppression of multiple death programs reinforce one another [7,29,30,31].

Proof-of-concept intervention studies suggest that this resistance can be therapeutically modulated. In a key study examining Fn persistence and antibiotic response, treatment with metronidazole reduced intratumoral Fn burden and suppressed growth of Fn-associated colorectal tumors in mice, consistent with the idea that eliminating Fn can partially restore treatment sensitivity [32]. However, because broad antibiotics can disrupt the wider microbiome, there is strong interest in more precise approaches. One notable example is phage-guided therapy. A lytic phage targeting Fn has been engineered to deliver irinotecan-loaded nanoparticles selectively to Fn-colonized tumors, enabling simultaneous bacterial depletion and improved chemotherapy response in CRC models [33]. Additional strategies aim to block Fn colonization itself. Because Fn enrichment in CRC is mediated by *Fap2* binding to tumor-expressed *Gal-GalNAc*, competitive inhibition of this interaction (e.g., with *Gal/GalNAc* analogs) has been proposed as a way to reduce tumor colonization and its downstream effects [28]. Immunization-based strategies have also been explored. For example, vaccination with Fn proteins such as *AhpC* reduced Fn intestinal burden in mouse experiments, supporting the concept of host-directed approaches to limit Fn carriage [34].

Fn’s relationship with immunotherapy is more complex and appears to be context dependent. On one hand, the immunosuppressive ecosystem associated with Fn—including inhibitory receptor engagement (e.g., *Fap2—TIGIT*) and dysfunctional T-cell states—would be expected to impair responses to immune checkpoint blockade [23,26]. Mechanistic evidence supports an immunotherapy-resistant pathway driven by microbial metabolites. Fn-derived succinic acid has been shown to suppress dendritic-cell *cGAS-STING* signaling, reducing type I interferon output and weakening *CD8*^+^ T-cell priming, and higher Fn/succinate has been associated with poorer anti-*PD-1* responses in translational models [35]. Fn can also contribute to tumor-promoting metabolic remodeling. For example, microbial formate has been implicated in exacerbating CRC progression in vivo, highlighting how metabolic crosstalk may influence immune tone and therapeutic susceptibility [36].

On the other hand, under certain conditions Fn may enhance checkpoint blockade efficacy. In MSS CRC—typically less responsive to immunotherapy—Fn colonization has been reported to promote a more inflamed microenvironment and increase *PD-L1* expression, and in a clinical cohort of advanced CRC treated with anti-*PD-1* therapy, Fn-positive tumors were associated with improved progression-free survival [8]. In experimental systems, Fn was shown to augment responses to *PD-(L)1* blockade, supporting the possibility that Fn-driven inflammation can sometimes shift MSS tumors toward a state that is more amenable to immunotherapy [8]. Taken together, current evidence indicates that Fn can either hinder or enhance immunotherapy outcomes depending on the balance between immune suppression (e.g., inhibitory receptor engagement and impaired antigen presentation) and immune activation (e.g., pro-inflammatory signaling that increases *PD-L1* and T-cell recruitment) [8,35].

Overall, Fn represents a compelling example of how the tumor-associated microbiome can influence therapy response through convergent effects on cancer cell stress programs and anti-tumor immunity. These data support translational efforts to (i) incorporate Fn or Fn-associated pathways as biomarkers of treatment response, and (ii) develop targeted interventions—such as phage-based approaches, adhesion blockade, or selective eradication—to improve therapeutic outcomes in Fn-colonized CRC [32,33].

## 4. Methodological Approaches for Mutational Signature Detection

### 4.1. Computational Approaches for Signature Detection

Modern mutational signature analysis relies on two complementary computational strategies: (1) de novo extraction of novel signatures and (2) assignment (refitting) of known signatures. De novo extraction aims to discover mutational patterns directly from genomic data without prior knowledge, whereas assignment methods quantify the contribution of established reference signatures (e.g., COSMIC signatures) in samples. Below, we outline each approach, common tools, and their methodological differences and trade-offs.

De novo mutational signature extraction approach seeks to uncover signatures from mutation catalogs ab initio. The predominant technique is non-negative matrix factorization (NMF), which simultaneously optimize two matrices—one representing the mutation pattern of each signature and another representing the exposure of each signature in each sample [21]. NMF was applied to large cancer cohorts to reveal the first catalog of mutational signatures. This laid the groundwork for automated tools like SigProfilerExtractor, which performs hierarchical NMF-based signature discovery and was used by the Pan-Cancer Analysis of Whole Genomes (PCAWG) consortium and others [37]. For example, a recent study of >800 CRCs employed SigProfilerExtractor with 500 NMF replicates to identify a repertoire of signatures, including two linked to colibactin exposure (SBS88 and ID18) [4]. An alternative extraction tool is SignatureAnalyzer which uses a Bayesian variant of NMF with automatic relevance determination to infer both the signatures and their number [37]. This probabilistic approach can guard against overfitting by pruning extraneous signatures and was used in some analyses to cross-validate NMF results. In practice, both SigProfiler (deterministic NMF) and SignatureAnalyzer (Bayesian NMF) yield similar outputs (a set of signature profiles and their sample-wise activities), but they differ in methodology. Notably, de novo extraction enabled the discovery of the colibactin-associated signature. Pleguezuelos-Manzano et al. exposed human organoids to *pks^+^ E. coli* and applied NMF-based extraction (via the MutationalPatterns R package) to uncover a distinct “SBS-*pks*” pattern, later catalogued as SBS88 and ID18 [16]. A strength of de novo discovery is the ability to find previously unknown mutational processes. However, it is computationally intensive and requires sufficiently large and diverse cohorts to separate signatures reliably. If a dataset is small or its mutation spectra are not heterogeneous, extraction may return composite or unclear signatures. In practice, any novel signature is typically compared against known reference signatures (e.g., COSMIC) by cosine similarity to assess whether it is truly new or a variant of an existing signature [37].

In contrast, reference assignment methods assume a predefined set of signature profiles and focus on estimating their contributions in each sample. Mathematically, this is a matrix decomposition where the mutation catalog *V* is approximated by the product of known signatures *W* (fixed) and exposures *H* (solved for each sample) [37]. Assignment is far less data-intensive than extraction—even a single tumor’s profile can be analyzed—and it provides a standardized way to compare signature burdens across samples and studies. A classic tool is deconstructSigs, an R package that finds the linear combination of known signatures that best reconstructs a tumor’s mutational profile using constrained non-negative least squares regression [38]. Similarly, MutationalPatterns (an R/Bioconductor toolkit) offers a signature refitting function that fits known signatures to a spectrum using non-negative least squares [39]. MutationalPatterns is versatile in that it can perform both unsupervised NMF extraction and supervised refitting. These and other tools (e.g., SigProfilerAssignment [40]) differ in their optimization algorithms and cut-offs for assigning signatures. Some use straightforward non-negative least squares (as in MutationalPatterns and SigProfilerAssignment) while others incorporate quadratic programming or iterative removal of negligible signatures. The choice of fitting algorithm and thresholds can influence results, and different tools may include or exclude borderline signature contributions depending on their stringency. To mitigate overfitting—the tendency to assign too many signatures to a sample—most pipelines filter out signatures that contribute only a tiny fraction. For example, many studies require a minimum exposure (e.g., >5% of mutations) for a signature to be considered present in a sample. This improves robustness, since unconstrained fitting can otherwise yield biologically implausible assignments (e.g., spurious signatures appearing in a cancer type where they do not truly occur). In practice, researchers often combine both approaches. A novel signature might first be identified via NMF, then a reference-fitting step is used to screen and quantify that signature across large cohorts. Indeed, Diaz-Gay et al. followed this strategy—after discovering colibactin-linked signatures de novo, they applied SigProfilerAssignment to thousands of CRC genomes to measure SBS88/ID18 exposure, revealing that these signatures are enriched in early-onset cases and show geographic variation [4,40]. In summary, de novo extraction is indispensable for discovering new mutational processes, whereas assignment methods trade discovery for sensitivity in detecting known signatures with high confidence in individual tumors. The choice depends on the analysis goal: to find new signatures or to faithfully detect known ones.

### 4.2. Whole-Genome vs. Whole-Exome Sequencing Data

WGS provides a far more comprehensive somatic mutation catalog than WES, which is critical for sensitive mutational signature analysis. WGS surveys the entire ~3 billion base genome, whereas WES targets only the ~1–2% that encodes proteins (the exons). Consequently, WGS uncovers orders of magnitude more mutations per tumor, including non-coding mutations that WES cannot detect. For example, a WGS study of MSS CRCs reported a median of roughly 12,000 single-nucleotide variants (SNVs) per tumor, whereas WES of the same tumors typically identify only a few hundred coding SNVs [4]. In other words, most somatic mutations (and thus mutational signature signal) lie outside the exome. Even within coding regions, WES may miss a fraction of mutations due to capture inefficiencies. One cross-platform comparison found that ~20% of high-confidence coding mutations were only detected by WGS, and that WGS had a more uniform coverage distribution and about 50% more total variation in exonic regions than WES (partly by rescuing mutations in extreme GC-content areas that exome capture often fails to cover) [41]. This higher mutation yield of WGS translates directly to greater power for signature discovery and resolution.

Regarding signature resolution and sensitivity, detecting a mutational signature—especially if its contribution to a tumor’s mutations is small—requires enough mutations to distinguish that pattern from noise. WGS data, with its larger mutation counts and more uniform genomic coverage, enables finer separation of multiple co-existing signatures, whereas WES often yields too few mutations to deconvolve all but the dominant signatures. A PCAWG benchmark analysis of 746 cancers sequenced by both WES and WGS illustrated that 76% of cases showed the same dominant mutational signature with both methods, and overall the WES vs. WGS signature profiles were highly correlated (mean Pearson ~0.9) [41]. However, in nearly one-quarter of cases the two data types did not agree on the top signature. These discrepancies typically involved weaker or uncommon signatures that WES failed to resolve due to limited mutation counts. Because WGS covers the genome more uniformly (avoiding the regional dropouts and GC biases that plague exome capture), it provides a more even sampling of the mutation spectrum. Exome sequencing is known to have reduced coverage in very GC-rich or GC-poor regions, and indeed WES may under-detect mutations in such regions. WGS avoids this pitfall with unbiased coverage, which is advantageous for accurately measuring the true mutation spectrum in a tumor. Furthermore, certain mutation types and contexts are better profiled with WGS, as whole genomes capture many indels in non-coding or repetitive DNA that WES would miss, enabling detection of indel-specific signatures alongside base-substitution signatures. In summary, the breadth and uniformity of WGS data confer higher sensitivity to low-prevalence signatures and sharper discrimination between similar signatures.

The power of WGS is especially evident for subtle signatures such as those induced by colibactin-producing bacteria. These microbiome-derived mutational patterns (such as SBS88 and ID18) typically constitute only a small percentage of mutations in an affected tumor (often on the order of 5–20%) [17]. With WGS, even a 5–10% signature can be confidently detected because it corresponds to hundreds of mutations spread across the genome, rising above statistical noise. In contrast, WES data yielding perhaps tens of coding mutations with that pattern may be too sparse for robust detection. Practically, WES might find only a few SBS88-consistent mutations (for example, one or two characteristic TT > TA substitutions in a driver gene like *APC*), whereas the corresponding WGS of that tumor reveals dozens or hundreds of additional substitutions and indel events that confirm the signature’s presence. Large-scale studies highlight this point that the colibactin-associated signatures SBS88 and ID18 went unrecognized in earlier exome-based colorectal cancer studies, but were uncovered by whole-genome projects that had the mutation breadth to expose these rare processes [4]. Thus, WGS has been instrumental in discovering and quantifying such microbiome-induced mutational signatures, whereas WES would likely miss many of these subtler imprints due to its limited scope.

Targeted DNA sequencing panels, while cost-effective and useful for detecting specific mutations, are generally not suitable for mutational signature analysis. Panels cover only a small set of genes (typically a few dozen to a few hundred genes, totaling only 0.5–2 Mb of sequence), yielding much fewer mutations than even WES. This severely limits statistical power for signature extraction or refitting—often there are simply too few mutations to robustly distinguish signatures from random noise. For instance, tumors needed to have ≥5 somatic SNVs to even attempt signature analysis from using multi-gene panels (~1–2 Mb), and ~13% of cases had to be excluded for failing this minimum [17]. Even among analyzable cases, indel-based signatures could not be assessed in ~84% of tumors because the panel yielded fewer than 5 indels in most samples. These figures highlight the inherent sparsity of panel data. In practice, only very strong signatures (such as the UV light signature in melanoma or the mismatch-repair deficiency signature in microsatellite instability (MSI)-high tumors) might be detectable with targeted panels, and even then, the panel must happen to cover the genomic sites where those mutations occur. Thus, while targeted panels play a valuable role in genotyping key driver mutations or therapeutic targets, they rarely capture enough of the mutational spectrum for signature analysis. By contrast, WES provides a broader survey and has been used in many studies to identify predominant signatures, but it may under-represent the full complexity of mutational processes in a tumor. WGS remains the gold standard for mutational signature detection thanks to its unbiased, high-yield mutation profiles across the genome. It maximizes mutation discovery—from coding to non-coding, SNVs to indels—thereby offering the highest sensitivity and resolution for dissecting mutational signatures, including those arising from exogenous exposures like the microbiome.

### 4.3. Detection Sensitivity and Thresholds

Detecting colibactin-linked mutational signatures in CRC requires sufficient mutation burden and careful thresholds to distinguish true signal from background noise. One key factor is sequencing scope, as whole-genome data provide orders of magnitude more mutations than whole-exome or targeted sequencing, greatly enhancing sensitivity. If the mutation count is too low, a signature can be missed (false-negative) or a few random mutations might be overinterpreted as a signature (false-positive). Empirical analyses indicate on the order of 100 mutations maybe needed to call mutational signatures robustly in a single sample [42]. For example, a CRC cell line genome with ~300 mutations was required to reach a stable signature solution in one benchmark, whereas high-coverage WGS of clinical tumors (with matched normals to filter artifacts) could detect signatures with as few as ~60–70 mutations. Thus, samples with very low mutation burdens (e.g., early-stage tumors or targeted-panel data) are at risk of failing to reveal rare signatures.

To ensure confidence in calling a signature, analysts apply minimum thresholds. A common practice is to ignore or “drop” signatures contributing only a tiny fraction of mutations (often below ~5%) to avoid spurious assignments. In a large CRC sequencing survey mentioned earlier, SBS88 was only considered “present” if it accounted for >10% of the tumor’s SBS mutations [17]. Tumors with fewer than five total SNVs were excluded entirely from signature analysis in that study, since any signature attribution in such low-mutation cases would be unreliable. Notably, the colibactin-associated indel signature ID18 poses an even greater detection challenge in limited data. In the targeted-panel analysis, 84% of tumors had <5 indels total—far below the minimum needed to discern and indel mutation pattern—and consequently ID18 could not be evaluated in those cases. Likewise, even WES often yields insufficient indel counts to call ID18 confidently, whereas WGS is usually required to detect that signature.

When such minimum criteria are not met, two outcomes can occur, false negative (the signature is truly present but goes undetected) or false positive (noise is misclassified as the signature). For example, in one analysis of >900 CRC exomes, initial signature fitting falsely flagged around 30 tumors as SBS88-positive, presumably due to chance occurrence of a few colibactin-like mutations. By applying more stringent criteria—focusing on specific colibactin mutation motifs and using a machine learning classifier for validation—the authors greatly reduced these false positives and achieved reliable detection of true SBS88 carriers [17]. In general, a random sprinkling of a few SBS88-type mutations (e.g., a couple of TT > TA changes) can be expected in any tumor by chance. Therefore, a minimum count or fraction is required before confidently assigning the SBS88/ID18 label. If that threshold is not reached, the case is usually deemed signature-negative to avoid overcalling. This cautious approach sacrifices some sensitivity (mildly exposed tumors may be missed) but is necessary for specificity. As a workaround in ultra-low mutation scenarios, researchers sometimes rely on known hallmark mutations as proxies for a signature. For instance, a recurrent *APC* splice-site mutation (c.835-8A>G) is strongly associated with colibactin exposure and has been used as a surrogate marker in panel-based analysis. Such proxies, however, only capture a subset of the signature’s events. Ultimately, empirical evidence suggests that reliable detection of SBS88 and ID18 in CRC requires a sufficiently high mutation burden (often tens to hundreds of relevant mutations, or a signature contributing well above a few percent of the profile), and that both the sequencing strategy and post-analysis cutoffs must be carefully chosen to balance sensitivity and specificity [17,42]. Key analytical considerations and practical thresholds for detecting colibactin-associated mutational signatures are summarized in Table 2.

### 4.4. Sample Quality and Technical Artifacts

Formalin-fixed paraffin-embedded (FFPE) tumor specimens—the standard for archived clinical samples—often introduce spurious mutations that can distort mutational profiles. During formalin fixation, DNA undergoes damage. Notably, hydrolytic deamination of cytosine to uracil causes artifactual C:G → T:A transitions, and oxidative lesions (e.g., 8-oxoguanine) lead to C:G → A:T transversions [43]. These technical artifacts can accumulate in poorly preserved samples. From the comparative study, five-fold more SNVs were called in FFPE DNA than in matched fresh-frozen DNA, even after error-correction, and moreover, ~92% of the FFPE-only variants were low-allele-frequency C > T changes characteristic of cytosine deamination damage. Such damage-induced mutations inflate the background mutation burden and can mimic real mutational patterns, complicating signature analysis.

Because mutational signature algorithms assume the input mutations are true somatic events, the inclusion of FFPE-induced artifacts confounds signature detection. False-positive “mutations” from DNA damage are especially problematic since they may be misinterpreted as genuine biological features. In fact, the aggregate “FFPE signature” of formalin damage closely resembles legitimate biological signatures. For example, the formalin-induced base-substitution profile is highly similar to COSMIC SBS30 (the signature of base-excision repair deficiency due to *NTHL1* mutation) and the ubiquitous age-related SBS1 (due to spontaneous 5-methylcytosine deamination) [44]. If these artifacts are not accounted for, computational deconvolution will miss-align mutational signatures. In practical terms, an archival CRC genome with abundant artifactual C > T mutations might erroneously appear enriched in ubiquitous endogenous signatures, while a subtle exposure-driven signature is masked. In summary, uncorrected FFPE-derived mutation catalogs can both mask true signature signals and produce false attributions of signatures.

This issue is especially pertinent when searching for the colibactin-associated signatures SBS88 and ID18 in clinical colorectal tumors. These bacterial toxin-induced signatures tend to be present at modest mutation counts per tumor, and they have distinctive sequence context biases (see Section 2.2) that set them apart from most endogenous processes [16,17]. However, in an FFPE sample the real colibactin fingerprint can be obscured by the noise of widespread C > T artifacts, and conversely artifact patterns could be mistaken for a colibactin signal if not properly filtered. A limited SBS88/ID18 signal may go undetected if dozens of spurious mutations raise the noise floor. Thus, sample-quality issues can lead to both false negatives and false positives in calling SBS88/ID18, emphasizing the need for diligent artifact mitigation when analyzing FFPE-derived genomes for mutational signatures.

To address FFPE artifacts, researchers employ both experimental and computational “de-artifacting” strategies. One the lab side, incorporating an uracil-DNA glycosylase (UDG) treatment during DNA library prep can excise uracil bases (created by cytosine deamination) before PCR amplification. This prevents many C > T errors being copied into sequencing libraries. It has been demonstrated that UDG pretreatment of FFPE DNA led to a dramatic reduction in artifactual C > T mutations, without impairing the detection of true mutations [45]. However, UDG cannot repair all damage. For instance, deamination of 5-methylcytosine produces a thymine (which is not recognized as foreign) and oxidative lesions like 8-oxo-dG are also unaffected. Therefore, additional measures are necessary. Bioinformatic artifact filters are critical post-sequencing. One approach is to statistically subtract the formalin-induced mutational signature from the tumor’s mutation catalog. For example, the algorithm FFPEsig was developed to computationally remove the characteristic FFPE mutation pattern, thereby unmasking the true biological signatures in the sample [44]. FFPEsig has been shown to enable accurate signature analysis in whole-genome FFPE samples by removing artifactual noise. A more granular tactic is variant-level filtering. FFPE artifacts tend to occur at low VAF and often at read termini (e.g., C > T changes at fragment ends), so applying VAF thresholds or filtering mutations with damage-associated sequence context can exclude many false calls. Simple filtering could remove the bulk of FFPE-specific variants (most of which were low-VAF C > T) [43], though overly strict cutoffs could also discard genuine subclonal mutations. Newer machine-learning classifiers improve on heuristic filters by learning to distinguish true mutations from artifacts using multiple features. For example, a model called DEEPOMICS-FFPE (a deep neural network) was trained on matched FF vs. FFPE exome data, and it successfully identified ~99.6% of artifactual variants while retaining ~87% of true variants, including those at a low allelic fraction (F1-score ~88% on held-out data) [46]. Such tools outperform traditional filters (like simply removing all low-frequency variants) by preserving more real mutations and targeting specific artifact signatures. Finally, standard practice includes sequencing a matched normal tissue or blood sample alongside the tumor. While a normal sample does not prevent FFPE damage in the tumor DNA, it serves to eliminate germline polymorphisms and can highlight recurrent technical artifacts (e.g., oxidation hotspots or cross-contamination) that might otherwise be interpreted as tumor-specific mutations.

By applying these combined strategies—enzymatic damage repair (UDG), computational artifact removal (FFPEsig and similar methods), stringent variant filtering, and matched normal comparison—researchers can greatly reduce FFPE-induced noise. This in turn increases the fidelity of mutational signature analysis in CRC and ensures that signatures like SBS88 and ID18, if present, are detected reliably even in FFPE specimens [44].

### 4.5. Translational and Clinical Considerations

Mutational signature analysis in CRC has clear translational potential [42]. By stratifying tumors according to their underlying DNA damage processes, mutational signatures can define etiologically distinct CRC subgroups that often have unique clinical or pathological features [17]. A prominent example is the subset of CRC characterized by the colibactin-associated mutational signature. At the population level, lifestyle and diet may modulate such impact of colibactin exposure. Notably, a Western-style diet has been linked to higher prevalence of *pks^+^ E. coli* and higher incidence of CRC characterized by colibactin signatures [47]. This suggests that dietary patterns could influence the microbiome in ways that either promote or mitigate colonization by colibactin-producing bacteria. It follows that dietary modifications might help reduce the risk of microbiome-driven CRC, although this remains to be validated.

Integrating mutational signature detection into routine clinical genomic profiling is becoming increasingly feasible. While WGS is the optimal platform for comprehensive signature analysis, many clinically relevant signatures can be detected via WES as well. Notably, some large clinical sequencing assays use extended gene panels that, if sufficiently broad, can capture mutational signatures in certain cases. For instance, in the multi-gene panel study of over 5000 CRCs, about 7.5% of tumors were identified as SBS88-positive under computational analysis [17]—demonstrating that even targeted sequencing can reveal a signature when enough mutations are present and appropriate algorithms are applied. As cancer genomic profiling becomes more routine, it is conceivable that mutational signature “readouts” will start to appear in diagnostic reports. Indeed, there is precedent in other malignancies. For example, mutational signatures of homologous recombination deficiency are already used to guide the use of *PARP* inhibitor therapies in breast and ovarian cancers [48]. Similarly, reporting a colibactin-associated signature in a CRC patient’s genomic report could alert clinicians to a possible microbiome-linked etiology and support considerations such as microbiome-targeted adjuncts or enrollment in trials of microbiome-modulating treatments. Ongoing advances in both laboratory and computational techniques are steadily improving our ability to detect and interpret mutational signatures, suggesting that signature-based diagnostics may soon become a valuable component of precision oncology.

## 5. Clinical Translation and Applications

### 5.1. Diagnostic Applications

The expanding evidence for microbiome involvement in CRC is motivating microbiome-informed diagnostics, particularly as adjuncts to established screening tools such as FIT-based stool testing and colonoscopy. While microbial biomarkers are not yet part of routine CRC screening guidelines, several assay formats—especially targeted stool qPCR and multi-marker microbial panels—have progressed to clinically meaningful performance evaluations and may help address limitations of blood- or hemoglobin-based stool tests (e.g., lesions with minimal bleeding).

A leading example is stool detection of Fn. In a clinical study, adding a stool Fn qPCR assay to FIT markedly improved CRC detection sensitivity (≈92% vs. ≈73% for FIT alone) without compromising specificity, and also improved advanced adenoma detection compared with FIT alone (≈39% vs. ≈15%) [49]. These results support a practical diagnostic concept that microbial markers can complement FIT by capturing non-bleeding or low-bleeding neoplasia that may otherwise be missed.

Beyond single-organism assays, multiple studies have evaluated stool panels that include Fn together with other CRC-associated microbial markers. In particular, stool detection of *pks* island-associated genes (e.g., *clbA* as a proxy for colibactin-producing *pks^+^ E. coli*) has been investigated as part of broader microbial marker sets and may improve discrimination when combined with *Fusobacterium* measures [50]. Such multi-marker approaches reflect the observation that tumor-associated dysbiosis is rarely driven by a single organism and that combined microbial signals often outperform individual markers.

A complementary “current-to-near-term” diagnostic direction is shotgun metagenomics-based classifiers. Meta-analyses across cohorts show that a reproducible stool microbiome signal can distinguish CRC from controls with clinically relevant accuracy, supporting the feasibility of microbiome-based risk models and test development [51,52]. Although metagenomic classifiers currently face barriers for widespread implementation (cost, standardization, and analytical pipelines), they provide an important benchmark as microbiome-derived signals can generalize across cohorts when carefully modeled and validated.

Finally, liquid biopsy extensions are being explored. Proof-of-concept studies indicate that circulating bacterial DNA differs between CRC/adenoma patients and controls, raising the possibility of minimally invasive microbiome-informed blood tests in the future [53]. These approaches remain investigational. But they illustrate a broader diagnostic trajectory, where combining conventional screening with microbial markers (stool or blood) and, where available, sequencing-based host signatures may enable more sensitive and more etiologically informative CRC detection.

### 5.2. Therapeutic Applications

Therapeutic strategies targeting the microbiome in CRC aim to (i) reduce tumor-promoting bacteria, (ii) restore protective microbial functions, and (iii) improve responses to standard therapies (chemotherapy, targeted therapy, immunotherapy). A key translational principle is that microbiome interventions may be most effective when applied with a tumor’s microbiome-linked features. For example, antibiotic depletion of tumor-associated bacteria has shown proof-of-concept activity in CRC models as previously mentioned.

Fecal microbiota transplantation (FMT) represents a more global microbiome remodeling strategy, with the goal of shifting the gut ecosystem toward an immune-permissive and therapy-responsive state. While the strongest clinical precedent for FMT-enhanced immunotherapy comes from melanoma, early-phase CRC efforts are emerging. A phase II study in refractory MSS metastatic CRC combined responder-derived FMT with anti-*PD-1* therapy (tislelizumab) plus fruquintinib and reported objective responses and high disease control rates that exceeded expectations for immunotherapy alone in this setting [54]. These data support continued investigation, while highlighting practical challenges in donor selection, standardization, and safety.

Probiotics and synbiotics (probiotics plus fermentable substrates) are being tested primarily to improve treatment tolerance and potentially modulate inflammation. In CRC patients receiving 5-FU-based chemotherapy, supplementation with *Lactobacillus rhamnosus* GG reduced severe diarrhea and improved gastrointestinal tolerability in a randomized study [55]. Although supportive-care benefits are the most immediate clinical application, probiotics also remain of interest as microbiome modulators. Importantly, strain choice dosing, and patient selection are critical, and probiotic strategies should be aligned with safety considerations in immunocompromised hosts.

Beyond these conventional interventions, several preclinical and emerging strategies aim for higher specificity and mechanistic precision. One approach is engineered bacterial therapeutics that localize to tumors and deliver immune modulators or biologics in situ. An engineered probiotic system capable of producing checkpoint blockade nanobodies within tumors enhanced anti-tumor immunity and tumor control in preclinical models, illustrating how synthetic biology may transform microbes into programmable anti-cancer agents [56]. Another approach is to pharmacologically neutralize microbial virulence factors rather than broadly depleting bacteria. For colibactin-driven carcinogenesis, small-molecule inhibition of the colibactin-activating peptidase *ClbP* suppressed genotoxic and pro-tumor effects of colibactin-producing bacteria in experimental systems, supporting a “toxin-blocking” therapeutic concept [57]. Such strategies could be particularly relevant for patients whose tumors or tissues show evidence of colibactin-linked mutagenesis, providing a rationale for precision microbiome intervention.

Overall, microbiome-based therapies in CRC are moving from associative observations toward actionable interventions. The field is converging on a practical translational roadmap: Identifying clinically meaningful microbiome-linked subgroups, applying targeted or ecosystem-level microbiome interventions, and evaluating whether these approaches improve standard-of-care outcomes or enable new prevention strategies. Continued trial development and biomarker-driven patient selection will be essential to safely integrate microbiome-based therapies into routine CRC management.

## 6. Remaining Challenges and Future Directions

### 6.1. Scientific and Technical Challenges

Despite the identification of microbial mutational signatures, the precise timing and causality of microbiome-induced mutations in CRC remain unclear. For example, colibactin-associated DNA lesions appear to be imprinted very early in life—possibly even during childhood—long before cancer onset. However, directly linking an early-life exposure to eventual tumor initiation is challenging. Most supporting evidence is correlative, and further research is needed to establish causation and clarify the molecular mechanisms by which genotoxins like colibactin contribute to CRC development.

Studying carcinogenic microbe–host interactions is constrained by current experimental models. In vitro gut organoid systems, while powerful, have practical shortcomings. Introducing live bacteria or toxins into the close organoid lumen often requires laborious microinjection, and each organoid’s unique size makes controlled dosing difficult. Standard organoids also lack immune and stromal components, and they may not support the prolonged, low-dose microbial exposures that occur in real life. In vivo models likewise offer only partial insights. Germ-free or colonized mouse models can recapitulate some microbe-driven tumorigenesis, but species differences and short lifespans limit their fidelity to human CRC. Notably, colibactin-producing *E. coli* accelerate intestinal neoplasia in mice and induce characteristic DNA damage in vitro, yet the overall impact of colibactin on the human tumor mutational landscape in vivo remains unclear. These model constraints make it difficult to fully probe microbiome-induced mutational processes and their timing in humans.

Identifying subtle mutation patterns from microbial exposures poses significant technical challenges. Mutational signature analysis typically requires a substantial number of somatic mutations to discern a signal with confidence. In many CRC samples—especially early lesions or whole-exome sequenced tumors—mutation counts are low, and the modest imprint of a microbe-driven process can be drowned out by endogenous “background” mutations. For instance, distinguishing the colibactin-associated signature SBS88 from the ubiquitous age-related SBS1 is difficult when only a few mutations are present, leading to missed detections or false assignments. Moreover, using an overly large reference signature catalog can exacerbate noise-fitting. Too many possible signatures increase the risk of spurious attributions (overfitting), whereas too few may cause true signals to be missed. These issues highlight the need for highly sensitive and specific analytic methods tailored to detect microbiome-induced mutational patterns.

The gut microbiome is enormously variable across individuals, which complicates the reproducible characterization of microbiome–genome interactions. Not all patients harbor relevant genotoxic bacteria, and even among carriers, bacterial activity can fluctuate over time. Indeed, there is often a disconnect between tumor genomic evidence of a past microbial influence and the microbiome profile at diagnosis—for example, tumors can bear the colibactin mutational signature even when no colibactin-producing bacteria are detectable in the specimen. This discordance suggests that transient early exposures (potentially years prior) may have caused mutations that persist in the cancer genome, even though the microbe is no longer present. Moreover, carriage rates of colibactin-producing strains differ by population. Such epidemiological variability makes it challenging to definitively link specific microbes to cancer across diverse cohorts—many commensal differences and co-factors can confound the association.

A further technical hurdle is the absence of standardization in bioinformatic pipelines for mutational signature detection. Multiple computational tools (over 30) exist for extracting or fitting signatures, each with different assumptions. Inconsistencies between analysis methods can lead to divergent results—one algorithm may call a “colibactin signature” in a tumor that another algorithm deems insignificant. This lack of a gold-standard approach hampers comparability between studies and can yield both false negatives (missed signatures due to low sensitivity) and false positives (overzealous fitting of noise). Efforts are underway to develop consensus frameworks and reporting guidelines, but until these are widely adopted, interpreting microbiome-induced mutational signatures will require caution. Standardized, sensitive pipelines are needed to confidently distinguish true mutation patterns caused by microbes from technical artifacts and ubiquitous background processes.

### 6.2. Clinical and Translational Barriers

Despite growing insights into the microbiome–genome axis in CRC, significant hurdles impede clinical translation of these findings. Diagnostic applications face a lack of standardization and consistency. Studies often use divergent sample processing methods and sequencing pipelines, leading to variability in microbial detection and biomarker profiles between cohorts [58]. This heterogeneity, compounded by differences in diet, antibiotic exposures, and host genetics, complicates reproducibility and hampers the development of universally accepted microbiome biomarkers [59]. Moreover, many proposed microbiome-based diagnostics lack prospective validation in diverse populations. Most data come from retrospective case–control studies, and few assays have been tested in large, multi-center trials to confirm their predictive value in real-world settings [58]. This paucity of validated biomarkers, combined with limited clinician familiarity, impedes integration of microbiome diagnostics into routine workflows. Practical issues—from cost-effectiveness to how new tests would fit into clinical pathways—remain unresolved. Harmonized protocols and outcome-driven studies are needed before microbiome assays can become part of standard CRC care.

Therapeutic translation of microbiome research in CRC is equally challenging. Safety and reproducibility are chief concerns for microbiota-modulating interventions. Approaches such as high-dose probiotics, FMT, or bacteriophage therapy have shown promise in modulating tumor growth and treatment responses, but their effects can be inconsistent and patient-specific [60]. Inter-individual differences in baseline microbiome composition and immune status mean that an intervention beneficial in one patient may be less effective in another, limiting the reproducibility of results [60,61]. Ensuring long-term safety is also critical—for instance, FMT carries risks of transmitting pathogens or antibiotic resistance genes, and even probiotics can cause opportunistic infection in vulnerable patients [60]. Rigorous donor screening, quality control in manufacturing, and monitoring for adverse events (such as horizontal gene transfer or emergent antibiotic resistance) are necessary to build clinician and regulatory confidence in these therapies [61]. Regulatory uncertainty remains another barrier, as agencies worldwide have yet to reach consensus on how to classify and approve microbiome-based therapeutics. For example, FMT is regulated as a biological drug in some jurisdictions but treated as a tissue or “transplant” in others, creating confusion in approval pathways. Similarly, the development of defined live biotherapeutic products (e.g., consortia of cultured strains) faces strict requirements for manufacturing consistency and proof of efficacy, yet existing frameworks are still evolving to accommodate these complex biological products.

Finally, there is broad agreement that large, controlled trials are needed to move the field forward. Both microbiome diagnostics and therapeutics must demonstrate clear clinical utility in Phase III studies—improving early detection, patient outcomes, or safety—to justify their adoption. Ongoing efforts, such as international consensus guidelines and multi-center collaborations, aim to standardize microbiome research practices and facilitate such trials [59]. In summary, translating microbiome–genome crosstalk into oncology practice will require overcoming methodological variability through standardization, validating tools in prospective cohorts, and addressing the safety and regulatory challenges of microbiome-based interventions. Coordinated, cross-disciplinary research—including robust biomarker development, patient stratification strategies, and careful risk management—is essential to bridge the gap from bench to bedside in CRC [62].

## 7. Conclusions

Mounting evidence has firmly established that certain gut microbes can imprint the CRC genome and modulate tumor behavior. Notably, colibactin leaves a distinctive mutational footprint in CRC genomes. These colibactin-associated mutations are not random passenger events, as they occur early in tumor evolution and can hit crucial driver genes. Intriguingly, these signatures are enriched in certain epidemiological settings—particularly in early-onset CRC and in populations with high CRC incidence—implicating early-life exposure to colibactin-producing bacteria as one factor in the rising CRC burden in younger adults [4]. Thus, the *E. coli pks* island exemplifies a mechanistic microbiome–genome interaction whereby a bacterial toxin generates permanent, oncogenic DNA alterations in the host.

In parallel, Fn has emerged as a key microbe influencing CRC progression and therapy response. Fn is known to accelerate tumor growth and invasiveness through multiple mechanisms [25]. This organism also modulates the tumor immune microenvironment—generally blunting anti-tumor immune surveillance—and has been correlated with inferior patient outcomes in some cohorts. Mechanistically, intratumoral Fn can drive chemoresistance [29]. On the other hand, Fn’s impact on immunity has a paradoxical upside in certain contexts, as data indicate that its presence can sensitize MSS CRC to immune checkpoint blockade [8]. These findings emphasize that microbial influences on tumor behavior are complex and may vary by bacterial strain, tumor context, and treatment modality.

From a translational perspective, these insights open new avenues for CRC risk assessment and intervention, while also posing challenges. Colibactin-linked mutational signatures in tumors serve as a molecular fossil record of past microbial exposure, defining a subset of CRC with unique etiology. For instance, identifying the “colibactin signature” in an early tumor might prompt consideration of past microbiota exposure as a contributing factor, and in the future may guide tailored preventive strategies. Stool-based assays for microbial biomarkers are being explored, as adding Fn DNA quantification to fecal immunochemical testing has already demonstrated improved detection of advanced neoplasia in screening populations. Similarly, gut metagenomic analyses might augment risk stratification by flagging high-risk microbial colonization.

Therapeutically, targeting cancer-associated microbes represents an innovative angle in CRC management. Proposed strategies include selective antibiotic or probiotic interventions, bacteriophage therapy, and even small-molecule inhibitors so neutralize microbial virulence factors. Recent studies have isolated lytic phages against Fn that can suppress *Fusobacterial* growth and attenuate CRC cell proliferation in vitro and in mouse models, laying groundwork for microbiome-targeted adjuncts to therapy [63]. Likewise, inhibitors of the colibactin pathway are under investigation [64]. Such interventions are intriguing, as they aim to disarm the oncogenic potential of the microbiota without necessarily eradicating the bacteria (thus potentially minimizing collateral disruption to the microbiome). Nevertheless, significant hurdles remain. The translational path for microbiome-based diagnostics and therapies is complex—encompassing issues of delivery, off-target effects, resistance, and regulatory approval. Moreover, the causal contributions of microbiota to human CRC progression need to be definitively proven in clinical settings, and patient selection (e.g., identifying who would benefit from microbiome-targeted therapy) is still an open question.

In summary, the interplay between certain microbes and the host genome has added a new dimension to our understanding of colorectal carcinogenesis. Bacterial factors like colibactin can create mutations that initiate tumors, while others like *Fusobacterium* can shape the tumor’s inflammatory and immune milieu, thereby influencing disease course and treatment efficacy. These discoveries emphasize a promising yet challenging frontier in CRC research. Ultimately, integrating microbiome insights into clinical practice—through improved screening tools, preventive measures, or adjuvant therapies—could enhance precision oncology for CRC. Achieving this will require further multidisciplinary research and carefully designed trials to overcome current gaps and ensure that manipulating the microbiome leads to tangible patient benefit.

## Figures and Tables

**Table 1:** Key evidence linking colibactin-producing *E. coli* to mutational signatures and colorectal carcinogenesis.

Study	Model/Cohort	Sequencing Platform	Key Findings	Significance for CRC
Nougayrede et al., 2006 [10]	Human cell lines infected with *pks^+^ E. coli*	Targeted assays, cytogenetics	*pks^+^ E. coli* induces DNA double-strand breaks and cell-cycle arrest	First demonstration of direct genotoxicity by colibactin
Arthur et al., 2012 [11]	Mouse models (Il10^−/−^, inflammation-associated CRC)	Targeted sequencing, pathology	*pks^+^ E. coli* promotes invasive CRC in vivo	Establishes tumor-promoting role of colibactin in vivo
Pleguezuelos-Manzano et al., 2020 [16]	Human colonic organoids exposed to *pks^+^ E. coli*	WGS	Identification of colibactin-associated mutational signatures (later SBS88/ID18)	Causal link between colibactin exposure and specific mutational signatures
Dziubanska-Kusibab et al., 2020 [5]	Human CRC tumors	WGS	Detection of SBS88/ID18 in human CRC genomes	First confirmation of colibactin signatures in patients
Lee-Six et al., 2019 [18]	Normal human colonic crypts	WGS	Early-life mutational imprints in normal epithelium	Supports early timing of colibactin exposure
Diaz-Gay et al., 2025 [4]	981 CRCs from 11 countries	WGS	SBS88/ID18 enriched in early-onset CRC and high-incidence regions	Links microbiome mutagenesis to molecular epidemiology
Chen et al., 2023 [19]	Human CRC and normal tissue	WGS, phylogenetic modeling	Truncal colibactin mutations in tumors and adjacent crypts	Demonstrates early, clonal imprinting
Terlouw et al., 2020 [20]	CRC patients with unexplained polyposis	WES + targeted validation	Recurrent *APC* splice-site mutation (c.835-8A>G) linked to SBS88	Connects colibactin signature to a specific driver mutation
Georgeson et al., 2024 [17]	Large CRC cohort (clinical sequencing)	Targeted panels/WES	Colibactin signatures associated with *APC*, *SMAD4*, *TP53* alterations and survival	Shows clinical relevance beyond WGS-only studies

**Table 2:** Methodological considerations and practical recommendations for detecting colibactin-associated mutational signatures (SBS88 and ID18).

Analytical Step	Recommended Practice	Minimum Criteria	Common Pitfalls	Notes/Examples
Sample type	Prefer fresh-frozen tissue; FFPE acceptable with artifact correction	Matched normal recommended	FFPE-induced C > T artifacts inflate background	FFPE samples require dedicated filtering (see below)
Sequencing platform	WGS preferred	≥60–100 total SNVs	WES or panels often underpowered	ID18 indel detection usually requires WGS
Mutation calling	High-specificity somatic variant callers with matched normal	VAF-aware filtering	Low-VAF artifacts misclassified as real mutations	Avoid aggressive VAF cutoffs that remove true subclonal events
Signature strategy	Reference-based fitting with restricted signature set	SBS88 ≥ 5–10% of SBS mutations	Overfitting with large signature catalogs	Limit reference signatures to CRC-relevant processes
De novo extraction	Use only in large, heterogeneous cohorts	≥100 tumors	Composite or unstable signatures in small datasets	Best for discovery, not single sample calling
SBS88 detection	Require characteristic A/T-rich sequence context	≥10–20 SBS88-consistent mutations	Chance occurrence of T > N mutations	Context validation improves specificity
ID18 detection	Assess indel spectrum separately	≥5–10 indels total	Insufficient indel counts in WES/panels	ID18 often missed without WGS
FFPE artifact control (lab)	UDG treatment	Pre-library enzymatic repair	Residual oxidative damage	UDG mainly reduces C > T deamination artifacts
FFPE artifact control (bioinformatics)	Explicit artifact removal or modeling	Artifact signature subtraction	Masking of true biological signatures	FFPEsig or ML-based classifiers recommended
Thresholding	Apply minimum contribution cutoffs	Typically, ≥5–10% exposure	False negatives for weak signatures	Conservative thresholds favored in clinical contexts
Proxy markers	Use hallmark driver mutations when data sparse	*APC*:c.835-8A>G present	Incomplete capture of signature burden	Useful for panel-based studies
Reporting standards	Report mutation counts, thresholds, and QC	Transparent methods section	Irreproducible results	Align with COSMIC/STORMS-stype reporting

## Data Availability

No new data were created or analyzed in this study. Data sharing is not applicable to this article.

## Abbreviations

The following abbreviations are used in this manuscript:

5-FU	5-fluorouracil
CRC	Colorectal cancer
ctDNA	Circulating tumor DNA
DSB	Double-strand break
FFPE	Formalin-fixed paraffin-embedded
FMT	Fecal microbiota transplantation
Fn	*Fusobacterium nucleatum*
ICL	Interstrand cross-link
MSI	Microsatellite instability
MSS	Microsatellite stable
NMF	Non-negative matrix factorization
OR	Odds ratio
PCAWG	Pan-Cancer Analysis of Whole Genome
SNV	Single-nucleotide variant
UDG	Uracil-DNA glycosylase
VAF	Variant allele fraction
WES	Whole-exome sequencing
WGS	Whole-genome sequencing
